# Risk Factors for Community Colonization With Extended-Spectrum Cephalosporin-Resistant Enterobacterales (ESCrE) in Botswana: An Antibiotic Resistance in Communities and Hospitals (ARCH) Study

**DOI:** 10.1093/cid/ciad259

**Published:** 2023-07-05

**Authors:** Ebbing Lautenbach, Mosepele Mosepele, Rachel M Smith, Ashley Styczynski, Robert Gross, Leigh Cressman, Anne Jaskowiak-Barr, Kevin Alby, Laurel Glaser, Melissa Richard-Greenblatt, Laura Cowden, Kgotlaetsile Sewawa, Dimpho Otukile, Giacomo M Paganotti, Margaret Mokomane, Warren B Bilker, Naledi Mannathoko

**Affiliations:** Division of Infectious Diseases, Department of Medicine, Perelman School of Medicine, University of Pennsylvania, Philadelphia, Pennsylvania, USA; Center for Clinical Epidemiology and Biostatistics, Perelman School of Medicine, University of Pennsylvania, Philadelphia, Pennsylvania, USA; Department of Biostatistics, Epidemiology, and Informatics, Perelman School of Medicine, University of Pennsylvania, Philadelphia, Pennsylvania, USA; Department of Internal Medicine, University of Botswana, Gaborone, Botswana; Division of Healthcare Quality Promotion, Centers for Disease Control and Prevention (CDC), Atlanta, Georgia, USA; Division of Healthcare Quality Promotion, Centers for Disease Control and Prevention (CDC), Atlanta, Georgia, USA; Division of Infectious Diseases, Department of Medicine, Perelman School of Medicine, University of Pennsylvania, Philadelphia, Pennsylvania, USA; Center for Clinical Epidemiology and Biostatistics, Perelman School of Medicine, University of Pennsylvania, Philadelphia, Pennsylvania, USA; Department of Biostatistics, Epidemiology, and Informatics, Perelman School of Medicine, University of Pennsylvania, Philadelphia, Pennsylvania, USA; Center for Clinical Epidemiology and Biostatistics, Perelman School of Medicine, University of Pennsylvania, Philadelphia, Pennsylvania, USA; Center for Clinical Epidemiology and Biostatistics, Perelman School of Medicine, University of Pennsylvania, Philadelphia, Pennsylvania, USA; Department of Pathology and Laboratory Medicine, University of North Carolina, Chapel Hill, North Carolina, USA; Department of Pathology and Laboratory Medicine, University Pennsylvania, Philadelphia, Pennsylvania, USA; Department of Microbiology, Public Health Ontario, Toronto, ON, Canada; Department of Laboratory Medicine and Pathobiology, University of Toronto, Toronto, ON, Canada; Center for Clinical Epidemiology and Biostatistics, Perelman School of Medicine, University of Pennsylvania, Philadelphia, Pennsylvania, USA; Department of Medicine, Botswana-University of Pennsylvania Partnership (BUP), Gaborone, Botswana; Department of Medicine, Botswana-University of Pennsylvania Partnership (BUP), Gaborone, Botswana; Division of Infectious Diseases, Department of Medicine, Perelman School of Medicine, University of Pennsylvania, Philadelphia, Pennsylvania, USA; Department of Biomedical Sciences, University of Botswana, Gaborone, Botswana; Department of Biomedical Sciences, University of Botswana, Gaborone, Botswana; Department of Biostatistics, Epidemiology, and Informatics, Perelman School of Medicine, University of Pennsylvania, Philadelphia, Pennsylvania, USA; Department of Biomedical Sciences, University of Botswana, Gaborone, Botswana

**Keywords:** antibiotic, resistance, colonization, extended-spectrum cephalosporin, low and middle income countries

## Abstract

**Background:**

The epidemiology of extended-spectrum cephalosporin-resistant Enterobacterales (ESCrE) in low- and middle-income countries (LMICs) is poorly described. Identifying risk factors for ESCrE colonization is critical to inform antibiotic resistance reduction strategies because colonization is typically a precursor to infection.

**Methods:**

From 15 January 2020 to 4 September 2020, we surveyed a random sample of clinic patients at 6 sites in Botswana. We also invited each enrolled participant to refer up to 3 adults and children. All participants had rectal swabs collected that were inoculated onto chromogenic media followed by confirmatory testing. Data were collected on demographics, comorbidities, antibiotic use, healthcare exposures, travel, and farm and animal contact. Participants with ESCrE colonization (cases) were compared with noncolonized participants (controls) to identify risk factors for ESCrE colonization using bivariable, stratified, and multivariable analyses.

**Results:**

A total of 2000 participants were enrolled. There were 959 (48.0%) clinic participants, 477 (23.9%) adult community participants, and 564 (28.2%) child community participants. The median (interquartile range) age was 30 (12–41) and 1463 (73%) were women. There were 555 cases and 1445 controls (ie, 27.8% of participants were ESCrE colonized). Independent risk factors (adjusted odds ratio [95% confidence interval]) for ESCrE included healthcare exposure (1.37 [1.08–1.73]), foreign travel [1.98 (1.04–3.77]), tending livestock (1.34 [1.03–1.73]), and presence of an ESCrE-colonized household member (1.57 [1.08–2.27]).

**Conclusions:**

Our results suggest healthcare exposure may be important in driving ESCrE. The strong links to livestock exposure and household member ESCrE colonization highlight the potential role of common exposure or household transmission. These findings are critical to inform strategies to curb further emergence of ESCrE in LMICs.

## Teaser

Extended-spectrum cephalosporin-resistant Enterobacterales (ESCrE) colonization prevalence was high in clinic and community settings in Botswana.

Independent risk factors for ESCrE included healthcare exposure, travel, livestock farming, and presence of an ESCrE-colonized household member.

The continued marked increase in extended-spectrum cephalosporin-resistant Enterobacterales (ESCrE) represents a global threat [1–3]. ESCrE, which primarily comprise organisms producing an extended-spectrum beta-lactamase (ESBL) or AmpC beta-lactamase, have been identified as 1 of the highest priority antibiotic-resistant pathogens by both the Centers for Disease Control and Prevention and the World Health Organization [4, 5]. In addition, they are associated with limited antibiotic treatment options and worse clinical outcomes [4, 6]. Although advances have been made in our understanding of the epidemiology of these organisms in high-income countries [7–9], limited data exist from low- and middle-income countries (LMICs) [10]. Most LMIC-focused studies have been limited because of inconsistent identification and susceptibility testing, reliance on only clinical specimens, and a primary focus on the hospital setting [11–16]. There are few data on colonization by ESCrE, especially within outpatient and community settings, despite that colonization is usually a prerequisite for infection and an important stage in pathogenesis [17–20].

Recent work by our group in several geographic regions in Botswana noted ESCrE colonization prevalence of 30.8% in clinic patients, 24.3% in adult community participants, and 26.2% child community participants [21]. Given this high prevalence of ESCrE colonization, and the important role of colonization as a reservoir for transmission and a precursor to clinical infection, identifying risk factors for ESCrE colonization is critical [22]. These data, from an LMIC, will be vital in developing and implementing successful global strategies to decrease ESCrE colonization and infection. The goal of this study was to determine risk factors for ESCrE colonization in clinic and community settings using an established patient cohort [21].

## METHODS

This work was part of the Antibiotic Resistance in Communities and Hospitals studies conducted across 6 countries to evaluate the population-based prevalence of colonization with clinically significant antimicrobial-resistant organisms. This study was reviewed and approved by the institutional review boards of the University of Pennsylvania, University of Botswana, and the Botswana Ministry of Health and Wellness.

### Study Sites

As part of a previously described large surveillance project [21], this study was conducted in the capital city and 2 semirural surrounding villages across 3 districts in Botswana: (1) Gaborone; (2) Mochudi; and (3) Molepolole. In these 3 locations, we enrolled participants at 2 outpatient clinics per region (6 total).

### Study Participants

Enrollment occurred from 15 January 2020 through 4 September 2020 but was paused from 2 April 2020 through 21 May 2020 because of a countrywide coronavirus disease 2019 (COVID-19) lockdown. Study participants included clinic patients and community participants. No participant was enrolled more than once.

#### Clinic Patients

Patients presenting for care at a participating clinic were randomly selected for participation. If a participant (aged ≥18 years) provided informed consent, he or she underwent rectal swab sampling (1 swab sample per participant) performed by a trained research nurse. Once 1 participant had been enrolled, the next participant was randomly selected from among those present in the clinic and offered participation at that time.

#### Community Participants

Each enrolled clinic patient was invited to refer 3 additional adults (aged ≥18 years) to the study. Among the 3 individuals referred, we encouraged referral of at least 1 household member and 1 nonhousehold member if possible. In addition, each adult (clinic patient or community participant) was requested to refer his or her children for participation. On arrival to the clinic, the referred community participant was consented and enrolled in a manner identical to that of a clinic patient. Adults provided consent for their own participation. Children younger than age 18 years had their consent completed by their accompanying adult. Finally, children between the ages of 7 and 17 years were also asked to complete a document of assent. We encouraged referred community participants to enroll within 2 weeks of the clinic patient's visit.

### Microbiological Evaluation

All rectal swab samples were collected using COPAN FecalSwabs [21]. Swabs were inoculated onto chromogenic media (CHROMagar ESBL) for preliminary identification of ESCrE. Further phenotypic identification and susceptibility testing was conducted using VITEK- MS matrix-assisted laser desorption/ionization-time of flight mass spectrometry (bioMerieux, Durham, NC) and a VITEK-2 AST-GN89 card (bioMerieux), respectively. ESCrE were defined as Enterobacterales demonstrating nonsusceptibility to ceftriaxone or ceftazidime [23]. This definition was designed to incorporate ESBL-producing Enterobacterales.

### Data Collection

All participants underwent comprehensive data ascertainment using a structured data collection instrument used by the research nurse at the time of enrollment and rectal sampling. Data collected included demographics, comorbid conditions, healthcare exposures (eg, hospital, clinic) in the prior 6 months, foreign travel in the prior 6 months (including location and duration of stay), and antibiotic use in the preceding 3 months (including specific agent). We also collected information on specific activities including growing/tending crops, tending of animals (eg, livestock, poultry, swine), site of animal tending (ie, home, farm), catching or handling of fish, and preparation of fish, chicken, or meat for consumption or sale. We also asked whether the participant handled, treated, or disposed of waste other than from the household. We further queried about the main source of drinking water and the main source of water for purposes other than drinking as well as the sanitation facilities at the home. In addition to the specific activities noted here, we also assessed general animal exposures and the frequency of exposure per week. Additionally, we assessed consumption of specific food groups (eg, meat, poultry, seafood, eggs) and the frequency of consumption. Finally, we assessed characteristics of the participant's household including household size, ages of household members, and whether other household members were also study participants.

### Data Analysis

Cases were defined as participants with Vitek-confirmed ESCrE colonization, whereas controls were defined as participants without ESCrE colonization. Controls were not matched to cases. In unadjusted comparisons of cases and controls, continuous variables were compared using the Student *t* test or Wilcoxon rank-sum test, and categorical variables were compared using the χ^2^ or Fisher exact test. For the adjusted analyses, multivariable logistic regression was used. Variables from bivariable analyses with *P* values <.20 were considered for inclusion in the final multivariable model. Variables were added based on biologic plausibility and collinear variables were excluded. We evaluated whether a statistical interaction was present between the type of participant (ie, clinic patient, community participant) and prior antibiotic use, healthcare exposure, presence of an ESCrE-colonized household member, and travel. We also evaluated potential interaction by the study period (ie, pre–COVID-19 lockdown vs post–COVID-19 lockdown). Variables were retained in the final model if they had a *P* value of <.05 in the multivariable model or based on a priori hypothesis. We adjusted for the clustering within household using the Huber-White variance adjustment [24, 25]. The strength of the association was measured using an odds ratio. A 95% confidence interval (CI) was also calculated for each effect estimate. All analyses were performed using STATA v.17 (StataCorp, College Station, Texas) and R (R Core Team, 2022).

## RESULTS

Of 3514 participants approached, 2000 (56.9%) participated; 849 (42.5%) were enrolled in the pre-lockdown period, whereas 1151 (57.5%) were enrolled in the post-lockdown period. Of the 2000 total enrolled participants, 959 (48.0%) were clinic patients, 477 (23.9%) were adult community members, and 564 (28.2%) were child community members. There were 1073 (53.7%) participants referred by another participant, and 725 (36.3%) participants were living in the same household as another participant.

Among all participants, the overall median (interquartile range [IQR]) age was 30 years (12–41) and 1463 (73.2%) were women. The age distribution was as follows: (1) aged <18 years = 568 (28.4%); (2) aged 18–65 years = 1383 (69.2%); (3) aged >65 years = 49 (2.5%); and (4) aged <5 years = 262 (13.1%)

Of 2000 participants, 555 (27.8%) were colonized with ESCrE; 488 (24.4%) were colonized with 1 ESCrE organism, 59 (3.0%) were colonized with 2 organisms, and 8 were colonized with 3 distinct organisms. Of 592 unique isolates (ie, multiple isolates representing the same organism in 1 individual only counted once), the specific organisms were: *Escherichia coli* (n = 491), *Klebsiella pneumoniae* (n = 75), *Citrobacter freundii* (n = 8), non-*freundii Citrobacter* (n = 8), *Enterobacter cloacae* complex (n = 7), *E. fergusonii* (n = 2), and *K. oxytoca* (n = 1).

ESCrE colonization by age group was as follows: (1) aged <18 years = 152 (26.7%); aged 18 to 65 years = 396 (28.6%); (3) aged >65 years = 8 (16.3%); and (4) aged <5 years = 71 (27.1%). ESCrE colonization was greater in clinic participants (292/959 [30.5%]) compared with community participants (263/1041 [25.3%]) (OR [95% CI] = 1.29 [1.06–1.58]; *P* = .01). ESCrE colonization also varied by geographic region: Gaborone (224/700 [32.0%]); Mochudi (189/698 [27.1%]); and Molepolole (142/602 [23.6%], *P* = .003).

In comparing baseline characteristics of cases and controls (Table 1), cases were more likely to have traveled outside the country in the preceding 6 months. Of the 42 participants who reported travel, 40 (95.2%) had traveled to South Africa and only 4 (9.5%) reported more than 1 trip outside the country in the preceding 6 months.

**Table 1.** Association of Participant Demographics, Clinical Characteristics and Healthcare Exposures With ESCrE Colonization

Variable	Cases (ESCrE+) (n = 555) N (%)	Controls (ESCrE–) (n = 1445) N (%)	OR (95% CI)	*P* value
General/household				
Age (median, IQR)^a^	29 (13.5–39.0)	30 (11.0–42.0)		.20
Woman	418 (75.31)	1045 (72.32)	1.17 (.93–1.47)	.19
At least 1 other household member ESCrE-colonized	46 (8.29)	78 (5.40)	2.00 (1.18, 3.42)	.007
Living in the same household as another participant	196 (35.31)	529 (36.61)	0.94 (.76–1.16)	.60
Median (IQR) other household members^a^	3.0 (2.0–5.0)	3.0 (2.0–5.0)		.22
At least 1 household member aged <18 y	376 (70.41)	1003 (72.42)	0.91 (.72–1.14)	.40
Travel outside the country in the past 6 mo	18 (3.22)	24 (1.66)	1.96 (1.00–3.81)	.04
Comorbidities				
Diabetes mellitus	8 (1.44)	27 (1.87)	0.77 (.30–1.75)	.57
Liver disease	0 (0.00)	0 (0.00)	N/A	N/A
Malignancy	1 (0.18)	2 (0.14)	1.30 (.02–25.06)	>.99
Chronic kidney disease	0 (0.00)	1 (0.07)	N/A	N/A
Hemodialysis	1 (0.18)	1 (0.07)	2.60 (.03–204.41)	.48
Cardiovascular disease	2 (0.36)	2 (0.14)	2.61 (.19–36.07)	.31
Respiratory disease	12 (2.16)	21 (1.45)	1.50 (.67–3.21)	.32
HIV	151 (27.21)	397 (27.47)	0.99 (.79–1.23)	.95
Hypertension	47 (8.47)	166 (11.49)	0.71 (.50–1.01)	.052
Antibiotic use				
Received at least 1 antibiotic in past 3 mo	43 (7.75)	100 (6.92)	1.13 (.76–1.66)	.56
Received 2 or more antibiotics in past 3 mo	14 (2.52)	20 (1.38)	1.84 (.85–3.87)	.08
Healthcare exposures—study participants				
Visited hospital to receive care	118 (21.26)	261 (18.06)	1.22 (.95–1.57)	.11
Visited clinic to receive care	400 (72.07)	1058 (73.22)	0.94 (.75–1.18)	.61
Visited hospital for reasons other than receiving care	82 (14.77)	154 (10.66)	1.45 (1.07–1.95)	.01
Visited clinic for reasons other than receiving care	167 (30.09)	426 (29.48)	1.03 (.82–1.28)	.78
Hospitalized for any reason	15 (2.68)	21 (1.46)	1.86 (.89–3.82)	.09
Healthcare exposures—household members				
Visited hospital to receive care	57 (10.27)	120 (8.30)	1.26 (.89–1.78)	.19
Visited clinic at least once	164 (29.55)	432 (29.90)	0.98 (.79–1.22)	.91
Visited hospital for reasons other than receiving care	23 (4.14)	54 (3.74)	1.11 (.64–1.87)	.70
Visited clinic for reasons other than receiving care	67 (12.07)	184 (12.73)	0.94 (.69–1.28)	.76

Unless otherwise noted, a 2-tailed Fisher exact test was used.

Abbreviations: CI, confidence interval; ESCrE, extended-spectrum cephalosporin-resistant Enterobacterales; HIV, human immunodeficiency virus; IQR, interquartile range; N/A, not available; OR, odds ratio.

Wilcoxon rank-sum test.

Cases were also significantly more likely to have another ESCrE-colonized participant living in the same household. Of the total 134 participants with at least 1 ESCrE-colonized participant in the household, 77 (57.4%) had 1 ESCrE-positive household member, 28 (20.9%) had 2 such household members, 14 (10.4%) had 3 such household members, and five (3.7%) had 4 or more such household members.

Antibiotic use in the preceding 3 months was also more common among cases (Table 1). The most commonly used antibiotics were amoxicillin (49/103 = 47.5%), metronidazole (34/103 = 33%), ceftriaxone (24/103 = 23%), penicillin (19/103 = 18%), and azithromycin (n = 18). Of those participants who did receive an antibiotic, the median (IQR) time from last antibiotic dose to enrollment was 33 (14–58.5) days.

Healthcare exposures among cases and controls are compared in Table 1. Although Table 1 shows the exposure as dichotomous (healthcare exposure yes/no), results from an assessment of number of exposures (ie, 3 or more visits in the past 6 months) did not yield substantively different results (data not shown). Animal exposures and food consumption of cases and controls are noted in Table 2. All animal exposures and food consumption were classified as any occurrence at least once in the past week. There was no substantive difference when alternatively assessing these variables by frequency of exposure in the past week. Table 3 compares water sources, sanitation facilities, and waste handling.

**Table 2.** Odds of ESCrE Colonization Among Study Participants According to Animal Exposure and Food Consumption

Variable	Cases (ESCrE+) (n = 555) N (%)	Controls (ESCrE–) (n = 1445) N (%)	OR (95% CI)	*P* value
Animal exposures				
Grow or tend crops	152 (27.39)	406 (28.09)	0.96 (.77–1.21)	.78
Tend livestock at home	86 (15.50)	276 (19.10)	0.78 (.59–1.02)	.07
Tend poultry at home	144 (25.94)	465 (32.18)	0.74 (.59–.92)	.007
Tend swine at home	13 (2.34)	33 (2.28)	1.03 (.49–2.02)	>.99
Tend livestock on a farm	119 (21.44)	261 (18.06)	1.24 (.96–1.59)	.09
Tend poultry on a farm	96 (17.30)	211 (14.60)	1.22 (.93–1.60)	.14
Tend swine on a farm	15 (2.70)	41 (2.84)	0.95 (.48–1.77)	>.99
Catch or handle fish	20 (3.60)	42 (2.91)	1.25 (.69–2.20)	.47
Prepare fish, chicken, or other meat for consumption or sale	271 (48.82)	746 (51.63)	0.89 (.73–1.09)	.27
Food consumption				
Meat consumption	420 (75.67)	1103 (76.33)	0.96 (.76–1.22)	.77
Poultry consumption	412 (74.23)	1121 (77.58)	0.83 (.66–1.05)	.12
Seafood consumption	126 (22.70)	282 (19.51)	1.21 (.95–1.54)	.12
Egg consumption	225 (40.54)	570 (39.45)	1.05 (.85–1.28)	.68
Cow’s milk consumption	388 (69.91)	1020 (70.59)	0.97 (.78–1.21)	.78
Goat’s milk consumption	20 (3.60)	53 (3.67)	0.98 (.55–1.69)	>.99
Other animal milk consumption	2 (0.36)	3 (0.21)	1.74 (.14–15.21)	.62
Yogurt consumption	206 (37.12)	489 (33.84)	1.15 (.94–1.42)	.17
Cheese consumption	63 (11.35)	115 (7.95)	1.48 (1.05–2.07)	.02
Fresh vegetable consumption	483 (87.03)	1275 (88.23)	0.89 (.66–1.22)	.49
Fresh fruit consumption	396 (71.35)	1062 (73.49)	0.90 (.72–1.12)	.34

All activities assessed for occurring at least once within the preceding week. All animal exposures and food consumption classified as any occurrence at least once in the past week. There was no substantive difference when alternatively assessing by frequency of exposure.

Abbreviations: CI, confidence interval; ESCrE, extended-spectrum cephalosporin-resistant Enterobacterales; OR, odds ratio.

**Table 3.** Odds of ESCrE Colonization Among Study Participants According to Water Source, Sanitation Facilities, and Waste Handling

Variable	Cases (ESCrE+) (n = 555) N (%)	Controls (ESCrE–) (n = 1445) N (%)	OR (95% CI)	*P* value
Main drinking water source				
Piped water into dwelling	123 (22.00)	342 (23.73)	0.91 (.71–1.15)	.44
Piped water into yard/plot	476 (85.15)	1225 (85.01)	1.01 (.76–1.35)	>.99
Public tap/standpipe	9 (1.61)	29 (2.01)	0.80 (.33–1.74)	.71
Storage tank	17 (3.04)	44 (3.05)	0.99 (.53–1.80)	>.99
Main water source for purposes other than drinking				
Piped water into dwelling	96 (17.17)	293 (20.33)	0.81 (.62–1.05)	.11
Piped water into yard/plot	491 (87.83)	1258 (87.30)	1.05 (.77–1.44)	.82
Public tap/standpipe	10 (1.79)	30 (2.08)	0.86 (.37–1.81)	.86
Storage tank	17 (3.04)	55 (3.82)	0.79 (.43–1.40)	.50
Sanitation facilities				
Flush or pour-flush to piped sewage system	152 (27.19)	410 (28.45)	0.94 (.75–1.17)	.62
Flush or pour-flush to septic tank	31 (5.54)	94 (6.52)	0.84 (.53–1.29)	.47
Flush or pour-flush to pit latrine	8 (1.43)	52 (3.61)	0.39 (.16–.83)	.008
Pit latrine with slab	334 (60.18)	926 (64.08)	0.85 (.69–1.04)	.11
Waste handling				
Handle, treat, or dispose of waste other than from household (at least once/week)	169 (30.45)	458 (31.70)	0.94 (.76–1.17)	.63

Abbreviations: CI, confidence interval; ESCrE, extended-spectrum cephalosporin-resistant Enterobacterales; OR, odds ratio.

In multivariable analysis, independent risk factors for ESCrE colonization were: (1) younger age; (2) presence of at least 1 other member of the household colonized with ESCrE; (3) recent foreign travel; (4) hospital exposure for reasons other than receiving care; and (5) tending livestock on a farm (Table 4). There was a borderline significant association between recent antibiotic use and ESCrE colonization. There was no significant interaction by type of participant (ie, clinic patient vs community participant) or by the study period (ie, pre- vs post–COVID-19 lockdown).

**Table 4.** Multivariable Analysis of Risk Factors for ESCrE Colonization in Community and Outpatient Study Participants Residing in Botswana

Variable	Unadjusted OR	Adjusted OR (95% CI)	*P* value
Age	−	0.99 (.98–.99)	.010
Woman	1.17	1.20 (.95–1.53)	.133
At least 1 other ESCrE-colonized household member	2.00	1.57 (1.08–2.27)	.019
Foreign travel	1.96	1.98 (1.04–3.77)	.037
Visited hospital for reasons other than receiving care in past 6 mo	1.31	1.37 (1.08–1.73)	.009
Use of >1 antibiotic in past 3 mo	1.84	1.86 (.92–3.76)	.085
Tending livestock on a farm in past 6 mo	1.24	1.34 (1.03–1.73)	.028

Abbreviations: CI, confidence interval; ESCrE, extended-spectrum cephalosporin-resistant Enterobacterales; OR, odds ratio.

In a secondary multivariable analysis, we assessed the presence of an ESCrE-colonized household member as a continuous variable rather than as a categorical variable. In this analysis, a greater number of ESCrE-colonized household members was associated with ESCrE colonization (adjusted odds ratio [95% CI] = 1.26 [1.02–1.55]; *P* = .029).

## DISCUSSION

We found high prevalence of ESCrE colonization (27.8%) in study participants overall, with rates in clinic patients somewhat higher than in community participants. Independent risk factors for ESCrE colonization included being a visitor to a hospital in the past 6 months, presence of at least 1 other member of the household colonized with ESCrE, recent travel outside the country, and tending livestock on a farm. There was a borderline significant association between recent antibiotic use and ESCrE colonization.

The association of ESCrE colonization with foreign travel is consistent with prior work demonstrating similar associations between foreign travel and ESCrE, including ESBL-producing Enterobacterales [26–28]. Although onset of traveler's diarrhea and the type of travel has been associated with ESCrE acquisition during travel [26, 28], we did not ascertain this information from study participants. Relatively few participants in our study reported recent foreign travel, likely because of the country-wide COVID-19 travel restrictions. During this lockdown, citizens were under a national “stay at home” order with severe restrictions on travel and limited availability of public transportation. This likely curtailed opportunities for acquisition of ESCrE via this mechanism suggesting our identified association may be an underestimate.

We also noted a strong association between tending livestock on a farm and ESCrE colonization. Traditionally, many people in Botswana keep livestock and rear cattle at cattle-posts and/or farms in their home villages or other villages. The practice of routinely visiting the cattle posts and farmlands and returning to the more urban or semiurban places of work and residence, may have a contributory factor in livestock-to-people transmission of ESCrE, especially given there are few local regulations on antibiotic use in livestock.

ESCrE organisms have been reported globally from cattle in both surveillance (fecal samples) and clinical cultures [29, 30] and may be driven at least in part by frequent use of extended-spectrum cephalosporins in livestock [31, 32]. Few data exist from African countries, however [29, 33]. The impact of ESCrE colonization in farm animals on human colonization and infection with these organisms remains an area of controversy with limited data [34, 35]. In particular, data from LMICs are scarce. One recent study of ESBL-producing Enterobacterales (ESBL-EB) carriage in pig and abattoir workers in Cameroon and South Africa noted that of 53 workers in Cameroon, 79% had hand cultures positive for ESBL-EB, whereas no South African workers had positive cultures [36]. Of 72 pig fecal samples, 42% were positive in South Africa compared with 100% positivity in Cameroon. Genotypic analysis showed 93 ESBL-EB strains differentiated into 14 distinct clusters. Several strains isolated in humans were highly related to those detected in pigs at the same abattoir [36]. On the contrary, a recent study conducted on Reunion Island that assessed human, wastewater, and livestock samples for ESBL-EB discovered that although ESBL-EB isolation in all samples was common, phylogenomic analyses demonstrated high diversity with little overlap of strains from different sources [37]. Given the lack of data from African sites specifically, defining the prevalence of ESCrE colonization among farm animals in Botswana and conducting genomic characterization of paired animal and human ESCrE strains would be invaluable in more clearly elucidating this potential link.

We also noted an independent association between ESCrE colonization and presence of another ESCrE-colonized individual (ie, another study participant) in the household. Furthermore, we found there was a 25% increase in odds of ESCrE colonization for each additional ESCrE-colonized household member. The role of household transmission in community ESCrE has not been elucidated despite that the home represents the location in which individuals spends the majority of their day. The importance of the household is emphasized by past small reports that have demonstrated between 8% and 37% of household members of patients with ESCrE infection are also colonized with an ESCrE [38]. Molecular characterization of ESCrE isolates would be invaluable in accurately defining the household transmission dynamics in explaining our demonstrated association. In addition, identifying more precisely the characteristics of the household, and the people who reside in it, that drive transmission will be critical in devising interventions to curtail household spread. Longitudinal studies of ESCrE colonization in households would be valuable in identifying transmission over time, both person-to-person transmission as well as potential shared sources of acquisition (eg, animal exposure, food consumption, water sources) [22].

Finally, exposure to a hospital setting was also noted to be independently associated with ESCrE colonization, consistent with several past reports [22]. Given the typically higher rates of antibiotic resistance in hospital settings, potential acquisition of ESCrE in such sites is highly plausible. Indeed, in our prior work in Botswana, colonization rates were significantly higher in hospital settings (ie, 42.6%) compared with rates of approximately 30% and 25% in clinic patients and community participants, respectively [21]. As an alternative explanation for our findings, it is also possible that ESCrE colonization might be driving healthcare utilization. In Botswana and many other LMICs, family members have a role as “caregivers” of hospitalized patients performing roles such as bathing, bringing meals, and feeding inpatients during family member hospitalization [39]. This cultural practice may contribute to transmission of resistant organisms between visitors and patients. It is also notable that hospital visits by family members and friends were severely restricted for part of the study period because of COVID-19 pandemic restrictions. Thus, the number of visits to the hospital among study participants is likely lower than would be expected. This potentially limited the opportunity for acquisition of ESCrE by hospital patients from visitors and vice versa. Additional research is needed to identify whether specific activities might be associated with ESCrE acquisition to better target interventions to limit transmission.

This study had numerous strengths, including a large sample size, innovative enrollment strategy, considerable geographic diversity, inclusion of both adults and children, and comprehensive data collection. There were also several potential limitations. First, approximately 50% of eligible subjects chose to enroll, raising the potential for selection bias. Second, clinical data were ascertained only via interview, raising the potential for recall bias. Finally, we included only 3 geographic regions in Botswana, potentially limiting the generalizability to other regions of the country or to other African countries.

In summary, we found numerous independent risk factors for ESCrE colonization. In particular, the strong association of ESCrE and household member colonization is important given limited data on household transmission in Africa. Critical next steps should include longitudinal assessment of ESCrE colonization in households, and genomic characterization of colonizing ESCrE to determine transmission dynamics in Africa. Similarly, our findings suggest risk factors that may be unique to Africa such as animal/farm practices and hospital visitation cultural practices that could be targets for novel interventions. These risk factor data hold great promise in informing the future development and testing of strategies to curb the further emergence of these concerning pathogens. Given the important role of colonization as a precursor to clinical infection, elucidating risk factors for ESCrE colonization will also be vital in developing strategies to reduce ESCrE infections.

## Notes

***Financial support.*** This work was supported by the Centers for Disease Control and Prevention (CDC) Broad Agency Announcement (BAA) FY2018-OADS-01 (Contract #75D30118C02919) (to E. L.). This work was also supported by a CDC Cooperative Agreement FOA #CK-20-004-Epicenters for the Prevention of Healthcare Associated Infections (to E. L.). This work was also supported by core services from the Penn Center for AIDS Research (CFAR), a National Institutes of Health–funded program (P30 AI 045008).

***Supplemental sponsorship***. This article appears as part of the supplement “The Evolving Challenges of Antibiotic Resistance in Low- and Middle-Income Countries: Priorities and Solutions,” sponsored by the U.S. Centers for Disease Control and Prevention, and Health Security Partners.
